# *In Vivo* Cytogenetic Studies on Aspartame

**DOI:** 10.1155/2010/605921

**Published:** 2010-06-20

**Authors:** Entissar S. AlSuhaibani

**Affiliations:** College of Science, King Saud University, P.O. Box 261002, Riyadh 11342, Saudi Arabia

## Abstract

Aspartame (a-Laspartyl-L-phenylalanine 1-methylester) is a dipeptide low-calorie artificial sweetener that is widely used as a nonnutritive sweetener in foods and drinks. The safety of aspartame and its metabolic breakdown products (phenylalanine, aspartic acid and methanol) was investigated *in vivo* using chromosomal aberration (CA) test and sister chromatid exchange (SCE) test in the bone marrow cells of mice. Swiss Albino male mice were exposed to aspartame (3.5, 35, 350 mg/kg body weight). Bone marrow cells isolated from femora were analyzed for chromosome aberrations and sister chromatid exchanges. Treatment with aspartame induced dose dependently chromosome aberrations at all concentrations while it did not induce sister chromatid exchanges. On the other hand, aspartame did not decrease the mitotic index (MI). However, statistical analysis of the results show that aspartame is not significantly genotoxic at low concentration.

## 1. Introduction

**Figure 1:**
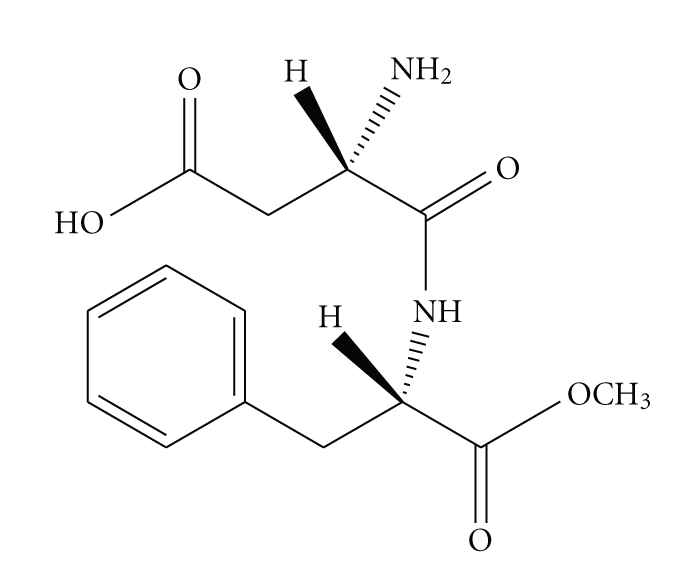
Structure of aspartame (N-l-alpha-aspartyl-l-phenylalanine 1-methylester).

Aspartame (a-Laspartyl-L-phenylalanine 1-methylester) is a dipeptide artificial sweetener, that is, widely used as a nonnutritive sweetener in food and drinks. Aspartame was discovered in 1965. In 1981, it became the first low-calorie sweetener approved by the Food and Drug Administration. The chemical structure and formula of aspartame is shown in Figure 1. 

Toxicological studies carried out with aspartame revealed that doses of aspartame required for harmful action would be very high and unrealistic [1]. Molinary [2] reported that aspartame was not mutagenic in Ames test. Also, aspartame was found not mutagenic in TA 100 and TA 98 Salmonella tester strains after nitrosation [3]. Moreover, It was not clastogenic *in vivo*, in mice [4]. And it was reported that aspartame did not induce DNA damage in rat hepatocytes [5]. Mukhopadhyay et al. [6] reported *in vivo* coexposure of aspartame and acesulfame potassium was negative for the induction of chromosome aberrations in male Swiss mice bone marrow cells. Although, Ishii [7] reported that aspartame does not cause brain cancer in rats. But Olney et al. [8], founded that aspartame might be associated with increased incidence of brain tumors. In addition, Gurney et al. [9], reported aspartame consumption in relation to brain tumor risk in children. Hence Butchko et al. [10] reviewed a study on safety of Aspartame and reported that it is safe. Whereas, Rencüzoğullar et al. [11], found that Aspartame induced chromosomal aberration at all concentrations (500, 1000 and 2000 *μ*g/mL) and treatment periods (24 and 48 h) in human lymphocytes dose dependently while it did not cause sister chromatide exchanges. In addition, Aspartame induced micronuclei at the highest concentrations only. The mitotic index was depressed in all used Aspartame concentrations. Whereas the replication index was decreased in the highest dose in the 48 h treatment. However Soffritti et al. [12, 13], reported evidence of Aspartame carcinogenicity (combined leukemia/lymphoma).

Bandyopadhyay et al., evaluated the genotoxic potential of of the low-dose range (7–37 mg/bwkg) of Aspartame by comet assay in the bone marrow cells of Swiss Albino mice. The comet parameters of DNA were increased in the bone marrow cells due to the sweetener-induced DNA strand breaks, as revealed by increased comet-tail extent and percent DNA in the tail [14].

The “acceptable daily intake” of aspartame, established by FDA, is 50 mg/kg; a food intake survey conducted by U.S. Department of Agriculture found some people in the U.S. consumed more than 16 mg/kg/day [15].

There have been multiple genotoxicity studies of aspartame each of which is inadequate for judging on its effect. The purpose of the present study was to determine whether and to what extent aspartame would induce chromosomal aberrations and sister chromatid exchanges. Bone marrow cells were chosen as indicator cells for their high sensitivity to clastogens [16].

## 2. Materials and Methods

The studies were conducted with male Swiss Albino mice aged 8–10 wk, weighing 20–25 g. Each experimental group consisted of five animals for each treatment and a control. They were maintained under conditions of ambient room temperature and relative humidity. The test substance aspartame was obtained from Sigma (Cat. no: A5139). Aspartame was dissolved in distilled water. Three concentrations: (i) 3.5 mg/kg body weight, (ii) 35 mg/kg body weight, and (iii) 350 mg/kg body weight were administered orally. These concentrations were selected according to cytotoxicity of aspartame. Control animals were treated with the solvent only. The animals were killed 24 hr later. Femoral bone marrow cells were analyzed for Chromosomal Aberration Assay (CA Assay), Sister Chromatid Exchange Assay (SCE Assay) and Mitotic Indices (MI).

Animals were intraperitoneally injected with BrdU 1.5 mg/g of body weight. 22 hours after BrdU injection animals were injected subcutaneously with 0.1 mL Colchicine solution (4 mg/10 mL distilled water)/10 g body weight. Two hours later, animals were sacrificed by cervical dislocation and both femurs were dissected to obtain bone marrow; bone marrow was obtained by injecting a phosphate-buffered saline solution into one end of the femur. Bone marrow cells were routinely processed by the standard procedure and slides were coded and stained in diluted Giemsa [17]. 50 metaphase cells were scored per animal. Chromatid and chromosome gaps, breaks, and rearrangements were evaluated in accordance with the method of Tice et al. [18]. The percentage of damaged cells (% DC) and chromosomal aberrations per cell (CA/cell) were calculated for each animal. Gaps were excluded from the analysis.

For the SCEs, slides were dried for at least 24 hours and stained using the fluorescence plus Giemsa method [19, 20]. SCE frequency was scored in 30 metaphases per mouse under second metaphases. The metaphases were examined at 1000× magnification. The results were used to determine the mean number of SCE (SCE/cell). In addition, The Mitotic Index (MI) was determined as the number of metaphases per 1000 cells from each animal. The MI explained the effects of aspartame on G2 stage of cell cycle.

Analysis of variance (ANOVA) was done to observe significant differences between the individual groups and Student's *t*-test was carried to compare the data of individual dose with that of the control. The level of significance was established at *P* = .01.

## 3. Result and Discussion

Table 1 presents data on the observation of chromosomal aberrations in the bone marrow cells of mice following exposure to aspartame. Aspartame induced a significant increase of chromosome aberration frequencies at concentrations 35 mg/kg body weight and 350 mg/kg body weight compared to concentrations 3.5 mg/kg body weight and control (Table 2). The aberrations were mainly chromatid in type.

However aspartame did not induce a significant increase of SCEs at any concentrations and treatment compared to control. Also, aspartame did not decrease the MI at any investigated concentrations for 24 hours treatment periods when compared with control (Tables 3 and 4).

In this study, Aspartame significantly induced CA at concentrations 35 mg/kg body weight and 350 mg/kg body weight. Nevertheless, aspartame did not induce the SCE or decreased the MI. While Rencüzoğullar et al., reported that Aspartame induced CAs at all concentrations (500, 1000 and 2000 mg/mL) and treatment periods (24 and 48 h) dose dependently, although it did not induce SCEs. However, Aspartame showed a cytotoxic effect by decreasing the MI at all concentrations and treatment periods dose-dependentl in human lyphocytes [11]. Furthermore Mukhopadhyay et al., found that treatment with different doses of Aspartame in combination with acesulfame-K induced weak clastogenic effects in the bone marrow cells of mice [6].

Aspartame is hydrolysed in the gut to yield aspartic acid, phenylalanine, methanol, and cyclised diketopiperazine [21, 22]. Shephard et al. [23], reported that Aspartame has a weak mutagenic effect after nitrosation. However, Butchko et al. [10], reported that Aspartame is safe and it was also reported that ASP was not mutagenic and clastogenic in animals [4, 5]. However, it must be taken into account that to determine the degree of disassociation of Aspartame as well as studying long-term accumulations in animals and humans.

According to these results, we can conclude that aspartame have a genotoxic risk. Therefore, it is necessary to be careful when using it in food and beverages as a sweetener.

## Figures and Tables

**Table 1:** Total chromosomal aberration in mouse bone marrow following exposure of aspartame.

Treatment	Chromosomal aberrations/250 cells					
(mg/kg body weight)	G′	G	B′	B	DC ± SD	CA/Cell ± SD
Control	5	0	5	0	0.02 ± 0.00	0.02 ± 0.00
3.5	3	0	5	0	0.02 ± 0.01^ns^	0.02 ± 0.00^ns^
35	7	1	8	1	0.04 ± 0.01**	0.04 ± 0.01**
350	8	0	11	0	0.05 ± 0.01**	0.05 ± 0.01**

G′, G = chromatid and chromosome gaps; B′, B = chromatid and chromosome breaks; DC = damaged cells with at least one CA (excluding gaps); CA = chromosomal aberrations; SD = standard deviation of the mean; * significant with both control and 3.5 mg/kg body weight at level 0.01; ^ns^ not significant with vehicle control.

**Table 2:** One-way ANOVA analysis of damaged cells showing differences among treatment groups.

Sources of variation	Degrees of freedom	Sum of squares	Mean sum of squares	*F* ratio
Among groups	3	203.350	67.783	21.866**
Within groups (errors, replicates)	16	49.600	3.100	

*Significant at level 0.01.

**Table 3:** Frequency of SCE and MI in mouse bone marrow following exposure of aspartame.

Treatment (mg/kg body weight)	Min-Max SCE	SCE/cell ± SE	MI ± SE
Control	0–17	3.20 ± 0.73	2.80 ± 0.37
3.5	3–10	4.60 ± 0.40	2.20 ± 0.37
35	3–15	4.40 ± 0.81	2.00 ± .0.55
350	3–20	5.40 ± 0.93	2.00 ± 0.32

A total 150 cells were scored for the SCE assay; 1000 cells from each animal were scored for MI.

**Table 4:** One-way ANOVA analysis of SCE and MI showing differences (if any) among treatment groups.

	Sources of variation	Sum of squares	DF	Mean square	*F*	Sig.
	Between groups	12.400	3	4.133		
SCE/cell	Within groups	44.400	16	2.775	1.489	0.255 (ns)
	Total	56.800	19			

	Between groups	2.150	3	0.717		
MI	Within groups	13.600	16	0.850	.843	0.490 (ns)
	Total	15.750	19			

*P*  > .05.
